# Influence of hepatic fibrosis and inflammation: Correlation between histopathological changes and Gd-EOB-DTPA-enhanced MR imaging

**DOI:** 10.1371/journal.pone.0215752

**Published:** 2019-05-13

**Authors:** N. Verloh, U. Probst, K. Utpatel, F Zeman, F. Brennfleck, J. M. Werner, C. Fellner, C. Stroszczynski, M. Evert, P. Wiggermann, M. Haimerl

**Affiliations:** 1 Department of Radiology, University Hospital Regensburg, Regensburg, Germany; 2 Department of Pathology, University Regensburg, Regensburg, Germany; 3 Center for Clinical Trials, University Hospital Regensburg, Regensburg, Germany; 4 Department of Surgery, University Hospital Regensburg, Regensburg, Germany; 5 Department of Radiology and Nuclear Medicine, Hospital Braunschweig, Braunschweig, Germany; Medizinische Fakultat der RWTH Aachen, GERMANY

## Abstract

**Objective:**

To evaluate the influence of an active inflammatory process in the liver on Gd-EOB-DTPA-enhanced MR imaging in patients with different degrees of fibrosis/cirrhosis.

**Material and methods:**

Overall, a number of 91 patients (61 men and 30 women; mean age 58 years) were included in this retrospective study. The inclusion criteria for this study were Gd-EOB-DTPA-enhanced MRI of the liver and histopathological evaluation of fibrotic and inflammatory changes. T1-weighted VIBE sequences of the liver with fat suppression were evaluated to determine the relative signal change (RE) between native and hepatobiliary phase (20min). In simple and multiple linear regression analyses, the influence of liver fibrosis/cirrhosis (Ishak score) and the histopathological degree of hepatitis (Modified Hepatic Activity Index, mHAI) on RE were evaluated.

**Results:**

RE decreased significantly with increasing liver fibrosis/cirrhosis (p < 0.001) and inflammation (mHAI, p = 0.004). In particular, a correlation between RE and periportal or periseptal boundary zone hepatitis (moth feeding necrosis, mHAI A, p = 0.001) and portal inflammation (mHAI D, p < 0.001) was observed. In multiple linear regression analysis, both the degree of inflammation and the degree of fibrosis were significant predictors for RE (p < 0.01).

**Conclusion:**

The results of this study suggest that the MR-based hepatic enhancement index RE is not only influenced by the degree of fibrosis, but also by the degree of inflammation.

## Introduction

Inflammation is an immune-mediated reaction to infections and foreign bodies. It can be distinguished into acute and chronic inflammation, which differ in duration, onset, and outcome. While acute inflammation can be treated reversibly, recurrent acute inflammation due to persistent acute infection may lead to tissue injury and destruction. Thereof, fibrosis results as a consequence of the pathophysiological response following tissue injury. However, the distinction between physiology and pathology is blurry as the right degree of inflammation and repair can heal a wound and restore tissue integrity and function, whereas excessive, uncontrolled inflammation or repair mechanisms may lead to tissue dysfunction. Inflammation may induce hepatocellular damage which will activate and promote hepatic stellate cells and Kupffer cells and finally leads to an inflammatory and fibrogenic response [1–3]. Consequently, the enhanced inflammatory and immune-mediated responses will promote additional hepatocyte necrosis and apoptosis, which triggers further fibrogenic processes [4]. By intervening in the signaling pathways that influence myofibroblast apoptosis, hepatic stellate cell inactivation, and extracellular matrix degeneration, hepatic fibrosis can be prevented or even reversed, it is therefore important to recognise inflammatory processes as early as possible in order to be able to take appropriate countermeasures.

Liver biopsy is the current gold standard for the diagnosis and evaluation of liver fibrosis, cirrhosis and inflammation in clinical practice. However liver biopsies are invasive procedures. They have poor patient compliance, are prone to sampling errors, and variability between observers. Moreover, they are associated with risks of complications such as infection and bleeding [5]. The absence of characteristic fibrotic septa and nodular configurations may impede histological diagnosis [6]. In addition, sampling errors may underestimate the severity of the disease [7, 8].

Heterogeneous hepatic parenchyma diseases may interfere with global liver function tests, e.g. the ICG-Test, as these tests cannot detect regional defects or hepatic compensation of them. Furthermore, an assessment of liver parenchyma using a dynamic imaging technique, such as ultrasound, is limited in terms of reproducibility [9]. Predictions on liver parenchyma may benefit from image-based tests for detecting regional and global liver changes. In recent years, liver-specific Gd-EOB-DTPA-enhanced MRI has evolved as a promising tool to assess liver function or stage liver fibrosis [10, 11].

In this study, the relationship between histopathological findings and the hepatic enhancement on Gd-EOB-DTPA-enhanced MRI were investigated to evaluate the effect of the hepatic inflammation status on contrast-enhanced signal intensity indices.

## Materials and methods

### Patients

**Fig 1:**
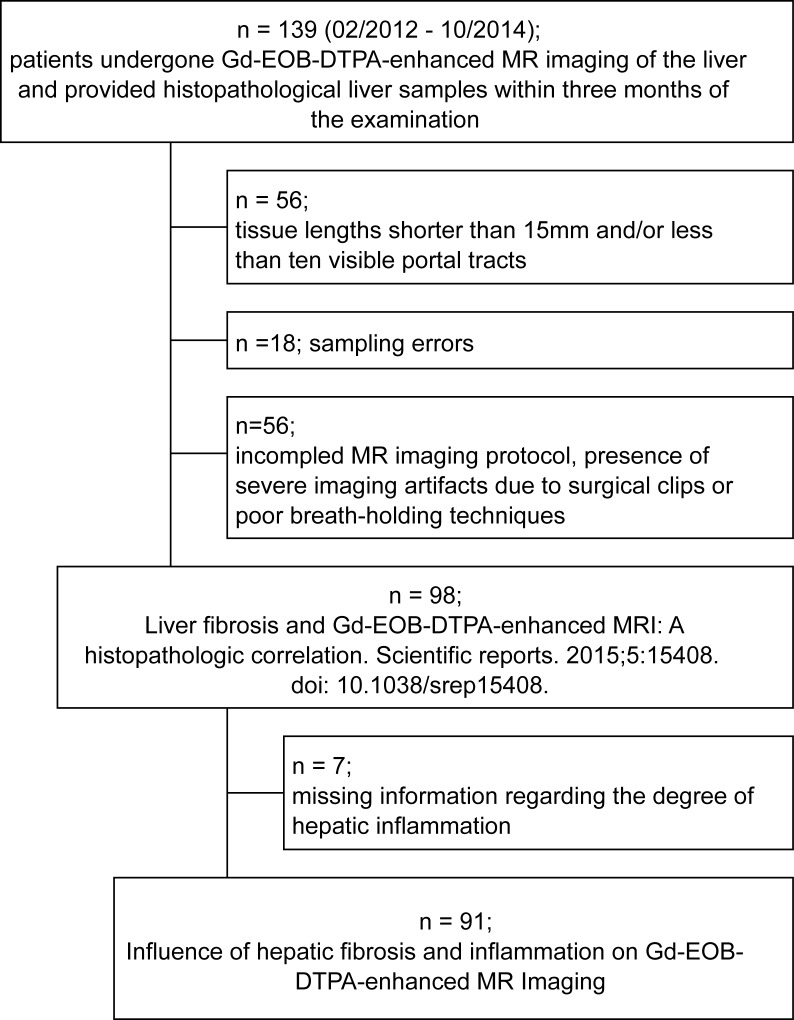
Flowchart of patient inclusion. The criteria for inclusion in this study were Gd-EOB-DTPA-enhanced MRI of the liver as well as evaluation of histopathological liver samples concerning fibrotic and inflammation findings (within three months of the examination) from a patient cohort analyzed in 2015.

A retrospective subgroup analysis was performed on a reported dataset over the correlation between liver fibrosis and Gd-EOB-DTPA-enhanced MRI, published October 2015 in Scientific Reports [12], Fig 1.

Approval from the local institutional review board of the University Hospital Regensburg was obtained, and this retrospective study was performed following the relevant guidelines and regulations. Written informed consent was obtained from all subjects.

Overall, 91 patients (61 men and 30 women; mean age, 58 years) were included in this retrospective study. The criteria for inclusion in this study were Gd-EOB-DTPA-enhanced MRI of the liver as well as evaluation of histopathological liver samples concerning fibrotic and inflammation findings (within three months of the examination). Detailed patient characteristics are given in Table 1.

**Table 1:** Baseline characteristics of the included patient cohort.

**parameters**	n = 91 (n = 65, liver resections; n = 35, needle biopsies)
**male**	61 (67.0%)
**age [years]**	58.21 ± 13.66
**height [m]**	1.72 ± 0.09
**weight [kg]**	81.2 ± 16.33
**BMI [kg/m^2^]**	27.17 ± 4.72
**modified HAI**	2.70 ± 2.19
**Ishak-Score**	2.27 ± 2.12
**Hepatocellular carcinoma**	24 (26.4%)
**Cholangiocellular carcinoma**	10 (11.0%)
**liver metastasis**	20 (22.0%)
**benign hepatic lesion**	6 (6.6%)
**Virus hepatitis**	20 (22.0%)
**Alcoholic liver disease**	17 (18.7%)
**biliary disease**	9 (9.9%)
**Nonalcoholic fatty liver disease**	7 (7.7%)
**Non-alcoholic steatohepatitis**	3 (3.3%)
**Autoimmune hepatitis**	1 (1.1%)
**Cardiac cirrhosis**	1 (1.5%)

The data are presented as mean (M) ± standard deviation (SD).

BMI: body-mass-index as a function of body weight to body height; Modified HAI: modified histologic activity index, based on findings of inflammation according to Ishak [14]; Ishak-Score: Histologic fibrosis index, based on fibrotic findings according to Ishak [14].

### MR imaging

All imaging was performed using a clinical whole-body 3-T system (MAGNETOM Skyra, Siemens Healthcare, Erlangen, Germany) with an 18-channel body matrix coil and a 32-channel spine matrix coil for signal perception. T1-weighted VIBE sequences with fat suppression and following MR settings were applied: repetition time (TR), 3.09 ms; echo time (TE), 1.17 ms; flip angle, 10°; parallel imaging factor, 2; slices, 64; reconstructed voxel size, 1.25 x 1.25 x 3.0 mm^3^; measured voxel size, 1.71 x 1.25 x 4.5 mm^3^; acquisition time, 14 sec. Images were acquired during breath-holding before, and 20 min after Gd-EOB-DTPA (Primovist®, Bayer Healthcare, Berlin) administration and every sequence covered the entire liver in the plain and the hepatobiliary phase (HBP).

The i.v.-injected Gd-EOB-DTPA dose was body weight adapted (0.025 mmol/kg body weight) and administered via bolus injection (flow rate, 1 mL/s), followed by 0.9% sodium chloride (20 mL).

### Image analysis

Signal intensity (SI) values were obtained by operator-defined region-of-interest (ROI) measurements of the liver (left liver lobe, 3 ROIs; right liver lobe, 3 ROIs) in T1-weighted VIBE images (before and after Gd-EOB-DTPA injection). The ROIs (circle shape, 1.0 cm^2^ - 3.5 cm^2^) were manually placed at identical locations in every sequence while excluding liver lesions, major branches of the portal and hepatic veins and imaging artifacts.

The following formula was applied to evaluate the liver function based on SI-values:

$$relative\;enhancement\;(RE)\;of\;signal\;intensity\;(SI)=\frac{mean({SI}_{HBP})-mean({SI}_{plain})\;}{mean({SI}_{plain})\;}$$

### Histopathological examination

The results of the histopathological samples were included if the report included fibrotic and inflammatory findings. All samples were fixed in formalin and embedded in paraffin for vertical microcuts (4 μm thickness). The cuts were mounted on glass slides, deparaffined with xylene and ethanol and stained with hematoxylin-eosin (HE) and Elastica van Gieson (EVG) according to standard protocols. Based on the specific staining (collagen, red; hepatocytes, yellow), the EVG was used to assess liver fibrosis. The samples were evaluated by two pathologists with professional expertise in liver histopathology. The readers scored independently of each other, while they were blinded for MR image analysis and patient data. In variation assessments, additional microscopic investigations were carried out jointly. Fibrosis and degree of inflammation were classified according to the Ishak Scoring System [13, 14].

### Statistical analysis

Simple linear regression models were used to determine the predictive power of SI-based indices in comparison to histopathological findings. Furthermore, a multiple linear regression model including Ishak Score and mHAI was calculated to investigate the additional variance elucidation of mHAI for the whole patient groups and based on parenchymal and tumorous diseases. Regression coefficients (B) with corresponding 95%-confidence intervals (95% CI) and the coefficient of determination (R^2^) are reported as effect estimates. The statistical significance level was set to 0.05 (two-sided), and all analyses were performed using SPSS software (version 24; IBM, Chicago, IL, USA).

## Results

### Histopathological findings

**Fig 2:**
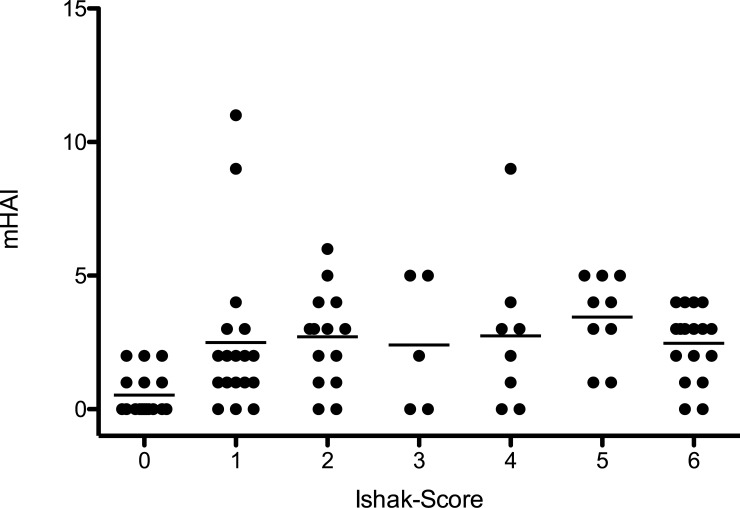
The level of inflammation plotted against the level of fibrosis/cirrhosis. The figure shows the level of inflammation, as characterized by the histologic activity index (mHAI) in comparison to the Ishak-Score. A dash indicates the mean of each group in the data cloud.

Eleven Patients showed no sign of hepatic fibrosis or inflammation. Six Patients presented a hepatic inflammation without an underlying fibrotic or cirrhotic liver parenchyma. Liver fibrosis/cirrhosis without hepatic inflammation occurred in eleven patients. Sixty-three patients showed an active inflammation in their liver fibrosis/cirrhosis (Table 2). The level of inflammation showed a significant correlation with the level of fibrosis/cirrhosis (p = 0.013, r = 0.260; Fig 2).

**Table 2:** Hepatic fibrosis in correlation to hepatic inflammation.

		*hepatic inflammation*	
		*mHAI = 0*	*mHAI ≥ 1*
***hepatic fibrosis***	*Ishak-Score = 0*	*11 (64.7)*	*6 (35.3)*
	*Ishak-Score ≥ 1*	*11 (14.9)*	*63 (85.1)*

The modified histologic activity index (mHAI) and the Ishak-Score as histologic fibrosis index based on Ishak [14] were used to evaluate the liver parenchyma. Data is presented as n with the percentage value within hepatic fibrosis.

In the case of liver fibrosis classification, 18.7% of the examined samples were free of fibrosis, while 62.6% showed different stages of fibrosis, and 18.7% had characteristics of cirrhosis (Table 3).

**Table 3:** Ishak-Score as histologic fibrosis index based on Ishak [14].

Ishak-Score	points	n	RE	SI(HBP)	SI(plain)
no fibrosis	0	17	1.13 ± 0.18	421.10 ± 71.21	199.15 ± 39.10
Fibrosis expansion of some portal areas, with or without short fibrous septa	1	18	0.89 ± 0.15	336.59 ± 60.57	177.86 ± 27.46
Fibrous expansion of most portal areas, with or without short fibrous septa	2	17	0.79 ± 0.17	313.64 ± 44.58	174.56 ± 26.46
Fibrous expansion of most portal areas with occasional portal to portal bridging	3	5	0.70 ± 0.10	292.60 ± 68.35	170.45 ± 30.70
Fibrous expansion of portal areas with marked bridging (portal to portal as well as portal to central)	4	8	0.56 ± 0.21	266.23 ± 53.24	169.13 ± 24.15
Marked bridging (portal-portal and/or portal-central) with occasional nodules (incomplete cirrhosis)	5	9	0.37 ± 0.14	257.34 ± 70.20	184.96 ± 34.67
Cirrhosis, probable or definite	6	17	0.26 ± 0.11	211.72 ± 51.77	166.50 ± 32.62

The data are presented as mean ± standard deviation (SD). Scoring system is based on histopathologic findings of collagen and hepatocytes. RE: relative enhancement as a function of SI-based indices of the hepatic parenchyma. SI: hepatic signal enhancement in plain or hepatobiliary (HBP) phase.

During hepatic inflammation different histopathological characteristics could be found, which should be analyzed separately. The sum of all criteria-points (A to D) then represents than the modified HAI (mHAI). In 73.6% of patient cases evaluated for mHAI A, no *periportal or periseptal interface hepatitis* was observed in the examined liver tissue samples. Only one samples showed a *confluent necrosis* (mHAI B), and 53.8% showed no sign of *focal lytic necrosis*, *apoptosis and focal inflammation* (mHAI C). Different stages of *portal inflammation* (mHAI D) could be observed in 65.9% of the examined samples (Table 4).

**Table 4:** Modified histologic activity index (mHAI) based on Ishak [14].

		points	n	RE	SI(HBP)	SI(plain)
**mHAI A: Periportal or periseptal interface hepatitis (piecemeal necrosis)**	None	0	67	0.80 ± 0.32	331.70 ± 83.34	184.75 ±31.97
	Mild (focal, few portal areas)	1	17	0.45 ± 0.27	245.07 ± 74.01	166.87 ± 33.612
	Mild / moderate (focal, most areas)	2	6	0.44 ± 0.23	221.97 ± 76.05	152.11 ± 31.77
	Moderate (continuous around < 50% of tracts or septa)	3	0	-	-	-
	Severe (continuous around > 50% of tracts or septa)	4	1	0.73	301.00	173.83
**mHAI B: Confluent necrosis**	None	0	90	0.71 ± 0.34	307.53 ± 90.13	178.43 ± 32.50
	Focal confluent necrosis	1	0	-	-	-
	Zone 3 necrosis in some areas	2	0	-	-	-
	Zone 3 necrosis in most areas	3	0	-	-	-
	Zone 3 necrosis + occasional portal-central bridging	4	0	-	-	-
	Zone 3 necrosis + multiple portal-central bridging	5	0	-	-	-
	Panacinar or multiacinar necrosis	6	1	0.76	345.33	196.17
**mHAI C: Focal (spotty) lytic necrosis, apoptosis and focal inflammation**	None	0	49	0.76 ± 0.34	324.62 ± 87.60	184.17 ± 32.82
	One focus or less per 10x objective	1	24	0.62 ± 0.33	288.81 ± 91.71	176.01 ± 32.82
	Two to four foci per 10x objective	2	15	0.73 ± 0.36	291.57 ± 91.72	167.14 ± 29.52
	Five to ten foci per 10x objective	3	1	0.30	175.80	135.23
	More than 10 foci per 10x objective	4	2	0.76 ± 0.03	318.12 ± 24.20	181.08 ± 10.25
**mHAI D: Portal inflammation**	None	0	31	0.91 ± 0.31	364.82 ± 90.36	190.08 ± 32.72
	Mild, some or all portal areas	1	27	0.76 ± 0.30	309.00 ± 65.30	176.28 ± 27.89
	Moderate, some or all portal areas	2	28	0.48 ± 0.26	250.01 ± 74.47	167.34 ± 33.39
	Moderate/marked, all portal areas	3	5	0.48 ± 0.20	274.18 ± 68.52	183.43 ± 31.56
	Marked, all portal areas	4	0	-	-	-

The data are presented as mean ± standard deviation (SD). Sum of all criteria-points represents the modified HAI. RE: relative enhancement as a function of SI-based indices of the hepatic parenchyma. SI: hepatic signal enhancement in plain or hepatobiliary (HBP) phase.

**Fig 3:**
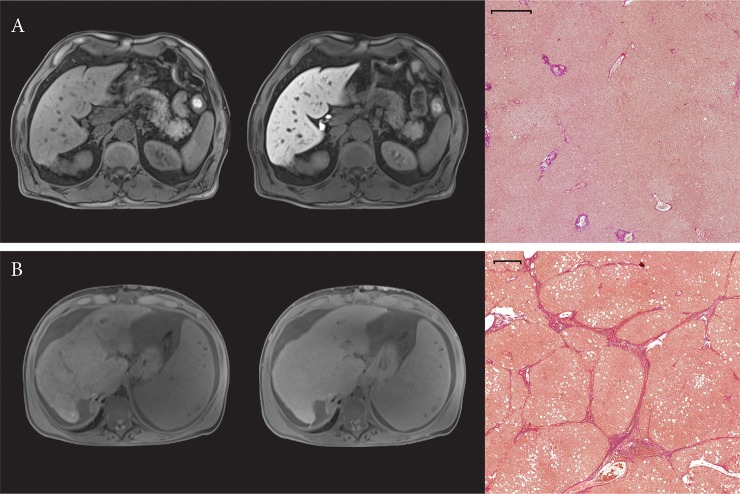
Representative images of a case with (A) high hepatic enhancement (RE: 1.29), absent inflammation (mHAI 0) and no fibrotic marker (ISHAK 0) is shown in comparison to a case with (B) low hepatic enhancement (RE: 0.30) paired with active inflammation (mHAI 9) and fibrosis (ISHAK 4). The first column represents the non-contrasted MR image while the second column represents the hepatobiliary phase. The corresponding histopathological imaging of the evaluated hepatic sections is shown in the third column. All displayed MR images have the same window and center level. The scale bar on histopathological images represents 500 μm.

In Fig 3 representative images for normal hepatic tissue without the presence of a fibrotic and inflammatory process (Fig 3A) and fibrotic hepatic tissue with inflammation (Fig 3B) are shown in addition to corresponding MR images. An increase of collagen tissue can be observed easily.

### Comparison of MRI-based hepatic enhancement indices to modified Histology Activity Index

**Fig 4:**
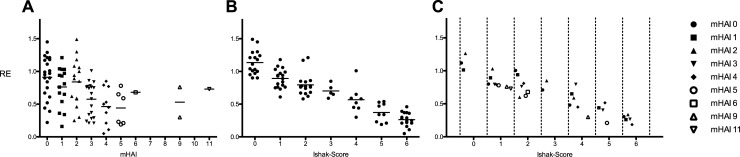
Relative enhancement (RE) values plotted with corresponding mHAI (A) and Ishak-Score (B) separately and combined (C). A dash indicates the mean of each group in the data cloud. Each dot represents the median of the combined model in graph C. The corresponding correlations with RE as the dependent variable are as followed: mHAI, r^2^ = 0.142, p = < 0.001; Ishak-Score, r^2^ = 0.792, p < 0.001. The combined model accounts for an R^2^ of 0.815.

Visual examination of the scatterplots revealed a linear relationship between mHAI and the MRI-based hepatic enhancement index relative enhancement (RE) (Fig 4A). Simple linear regression analyses (Table 5) showed significant correlations of mHAI to the tested MRI-based hepatic enhancement indices RE (r^2^ = 0.142; p = < 0.001), SI_HBP_ (r^2^ = 0.166; p = < 0.001) and SI_nativ_ (r^2^ = 0.072; p = 0.010).

**Table 5:** Simple linear regression models of SI-based indices with HFI and modified HAI.

			B (95% CI)	r^2^	p-value
RE	Ishak-Score		- 0.14 (- 0.15; - 0.12)	0.792	**< 0.001**
		liver resection	- 0.13 (- 0.15; - 0.11)	0.768	**0.010**
		needle biopsy	- 0.15 (- 0.17; - 0.12)	0.818	**0.012**
	mHAI		- 0.06 (- 0.09; - 0.02)	0.142	**< 0.001**
		liver resection	- 0.09 (- 0.13; - 0.05)	0.267	**< 0.001**
		needle biopsy	- 0.04 (- 0.09; - 0.01)	0.086	0.087
SI_HBP_	Ishak-Score		- 30.20 (- 36.04; - 24.36)	0.543	**< 0.001**
		liver resection	- 32.38 (- 40.74; - 24.02)	0.528	**< 0.001**
		needle biopsy	- 27.65 (- 36.22; - 19.01)	0.566	**< 0.001**
	mHAI		- 17.27 (- 25.41; - 9.12)	0.166	**< 0.001**
		liver resection	- 25.69 (- 37.72; - 13.66)	0.253	**0.001**
		needle biopsy	- 11.79 (- 23.27; - 0.31)	0.117	**0.044**
SI_plain_	Ishak-Score		- 3.55 (- 6.58; - 0.53)	0.058	**0.022**
		liver resection	- 5.50 (- 9.67; - 1.34)	0.115	**0.011**
		needle biopsy	- 1.63 (- 6.27; 3.01)	0.015	0.479
	mHAI		- 4.11 (- 7.21; - 1.01)	0.072	**0.010**
		liver resection	- 5.85 (- 10.66; - 1.03)	0.100	**0.018**
		needle biopsy	- 2.81 (- 7.08; - 1.47)	0.051	0.190

RE: relative enhancement as a function of SI-based indices of the hepatic parenchyma. SI: hepatic signal enhancement in plain or hepatobiliary (HBP) phase. mHAI: modified histologic activity index, based on findings of inflammation according to Ishak [14]: Ishak-Score: Histologic fibrosis index, based on fibrotic findings according to Ishak [14]. r^2^: coefficient of determination. p-value: level of significance.

In a subgroup analysis regarding the influence of the tissue sample we found no significant correlation of mHAI to the tested MRI-based hepatic enhancement index RE for needle biopsies whereas significant correlations could be observed for liver resections. In these subgroups the coefficient of determination (r^2^) increased for each tested value: RE, r^2^ = 0.267, p = < 0.001; SI_HBP_, r^2^ = 0.253, p = 0.001; SI_nativ_, r^2^ = 0.100, p = 0.018 (Table 5).

Regarding the subgroups of the overall mHAI the best coefficient of determination values was observed for mHAI D (*portal inflammation*) in relation to RE (r^2^ = 0.278; p < 0.001) and SI_HBP_ (r^2^ = 0.244; p < 0.001; Table 6). A subgroup analysis of the investigated tissue was not possible due to the small sample size.

**Table 6:** Simple linear regression models of SI-based indices with modified HAI and specific criteria of modified HAI (mHAI).

			B (95% CI)	r^2^	p-value
RE	mHAI		- 0.06 (- 0.09; - 0.02)	0.142	**< 0.001**
		mHAI A	- 0.17 (- 0.26; - 0.07)	0.123	**0.001**
		mHAI B	- 0.01 (- 0.10; 0.12)	0.000	0.785
		mHAI C	- 0.03 (- 0.10; 0.05)	0.007	0.419
		mHAI D	- 0.19 (- 0.26; -0.13)	0.278	**< 0.001**
SI_HBP_	mHAI		- 17.27 (- 25.41; - 9.12)	0.166	**< 0.001**
		mHAI A	- 47.26 (- 72.05; - 22.47)	0.139	**< 0.001**
		mHAI B	6.30 (- 23.71; 36.31)	0.002	0.678
		mHAI C	- 16.11 (- 36.05; 3.83)	0.028	**0.017**
		mHAI D	- 47.45 (- 65.05; - 29.85)	0.244	**< 0.001**
SI_plain_	mHAI		- 4.11 (- 7.21; - 1.01)	0.072	**0.010**
		mHAI A	- 11.93 (- 21.24; - 2.62)	0.068	**0.013**
		mHAI B	2.96 (- 7.87; 13.78)	0.003	0.589
		mHAI C	- 6.56 (- 13.73; 0.60)	0.036	0.072
		mHAI D	- 7.94 (- 15.05; - 0.83)	0.052	**0.029**

RE: relative enhancement as a function of SI-based indices of the hepatic parenchyma. SI: hepatic signal enhancement in plain or hepatobiliary (HBP) phase. mHAI: modified histologic activity index, based on findings of inflammation according to Ishak [14]: mHAI A: Analysis of periportal or periseptal interface hepatitis (piecemeal necrosis); mHAI C: Analysis of focal (spotty) lytic necrosis, apoptosis, and focal inflammation; mHAI D: Analysis of portal inflammation. r^2^: coefficient of determination. p-value: level of significance.

### Comparison of MRI-based hepatic enhancement indices to Ishak-Score

The scatterplot revealed a stringent linear relationship between the MRI-based hepatic enhancement index RE and the Ishak-Score (Fig 4B). The linear regression models of fibrotic markers on the signal intensity-based indices showed high coefficients of determination with significant character for RE (r^2^ = 0.792; p < 0.001) and SI_HBP_ (r^2^ = 0.543; p < 0.001; Table 5).

In contrast to the results of the subgroup analysis of the comparison of MRI-based hepatic enhancement indices to modified Histology Activity Index the coefficient of determination decreased in case of liver resection for RE, r^2^ = 0.768, p = 0.010 and SI_HBP_, r^2^ = 0.528, p < 0.001. No significant correlation could be observed regarding the influence on SI_nativ_ for needle biopsies (p = 0.160), while still a significant correlation for liver resection (p = 0.011) was observed.

### Multiple linear regression analysis of MRI-based hepatic enhancement indices to histopathological findings

In a multiple linear regression analysis, the effect of the Ishak-Score and mHAI were tested towards RE (Table 7). Both independent variables showed to be significant predictors of RE values (R^2^ = 0.815; Table 7, Fig 4C).

**Table 7:** Multiple linear regression analysis with the relative signal change (RE) as a dependent variable. The model showed a coefficient of determination (R^2^) of 0.815.

Independent variable	B (95% CI)	p-value
Ishak-Score	- 0.13 (- 0.15; - 0.12)	**< 0.001**
modified HAI	- 0.03 (- 0.04; - 0.01)	**0.001**

### Influence of hepatic inflammation (mHAI) on parenchymal enhancement for patients with known parenchymal and tumor diseases

**Fig 5:**
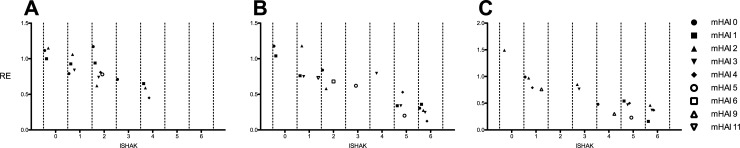
Relative enhancement (RE) values plotted against the combined model for patients with unknown/non-parenchymal disease (A), known inflammatory- (B), and non-inflammatory-parenchymal diseases (C). Each dot represents the median of the combined model. The corresponding correlations with RE as the dependent variable are as followed: A, R^2^ = 0.503; B, R^2^ = 0.849: C, R^2^ = 0.812.

In a multiple linear regression analysis, the effect of the Ishak-Score and mHAI were tested towards RE in patients who with unknown/non-parenchymal disease, known inflammatory-, and non-inflammatory-parenchymal disease (Table 8, Fig 5). For patients with unknown/non-parenchymal disease (Fig 5A, R^2^ = 0.503) the dominant factor was the liver fibrosis score (p ≤ 0.001), while the inflammatory showed no additional significant further variance elucidation (p = 0.319). The mHAI remained a significant influence factor in patients with known inflammatory-parenchymal disease (Fig 5B, p = 0.022, R^2^ = 0.849), as well as in patients with non-inflammatory-parenchymal disease (Fig 5C, p = 0.027, R^2^ = 0.812).

**Table 8:** Enhancement values for patients with known parenchymal and tumor diseases and their corresponding mHAI (Modified histologic activity index) and Ishak-Scores.

		modified HAI	Ishak-Score	SI (plain)	SI (HBP)	RE
**unknown/non-parenchymal disease**		1.27 ± 1.44	1.27 ± 1.28	188.45 ± 36.28	363.65 ± 91.88	0.92 ± 0.24
**Inflammatory disease**		2.79 ± 2.27	3.85 ± 2.31	169.53 ± 28.11	261.71 ± 75.96	0.54 ± 0.35
	Virus hepatitis	3.30 ± 2.41	4.25 ± 1.97	169.51 ± 27.25	254.52 ± 64.32	0.50 ± 0.28
	Biliary disease	2.00 ± 2.24	2.56 ± 2.65	171.20 ± 22.41	290.41 ± 92.95	0.69 ± 0.48
	Autoimmune hepatitis	2	6	195.17	244.00	0.25
	Nonalcoholic steatosis hepatis	2.00 ± 1.00	4.33 ± 2.89	156.08 ± 54.46	229.43 ± 111.85	0.42 ± 0.29
**Non-inflammatory disease**		2.92 ± 2.24	3.08 ± 1.98	177.66 ± 29.57	295.47 ± 62.17	0.67 ±0.30
	Alcoholic liver disease	3.12 ± 1.97	3.53 ± 2.00	185.20 ± 29.49	298.03 ± 63.56	0.61 ± 0.24
	Nonalcoholic fatty liver disease	1.57 ± 1.13	1.86 ± 1.57	165.41 ± 23.28	306.33 ± 46.30	0.88 ± 0.35
	Cardiac cirrhosis	9	4	135.23	175.80	0.30
**non-tumorous disease**		3.06 ± 2.18	4.13 ± 2.08	172.80 ± 32.41	259.67 ± 81.01	0.49 ± 0.32
**malignant tumorous disease**		2.04 ± 2.01	2.17 ± 1.86	179.31 ± 30.51	323.64 ± 78.61	0.80 ± 0.27
	Hepatocellular carcinoma	2.42 ± 1.77	3.29 ± 1.92	171.63 ± 30.17	284.46 ± 67.60	0.66 ± 0.26
	Cholangiocellular carcinoma	1.70 ± 1.25	1.70 ± 1.70	187.50 ± 32.74	345.08 ± 32.74	0.84 ± 0.25
	liver metastasis	1.75 ± 2.53	1.05 ± 0.89	184.43 ± 29.14	359.93 ± 71.12	0.95 ± 0.20
**Benign tumorous disease**		0.33 ± 0.82	0.17 ± 0.41	202.58 ± 42.38	416.18 ± 93.96	1.07 ± 0.32

The data are presented as mean ± standard deviation (SD). Sum of all criteria-points represents the modified HAI. RE: relative enhancement as a function of SI-based indices of the hepatic parenchyma. SI: hepatic signal enhancement in plain or hepatobiliary (HBP) phase.

**Fig 6:**
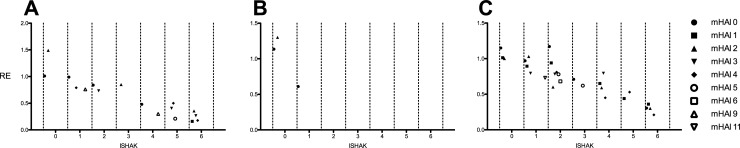
Relative enhancement (RE) values plotted against the combined model for patients with non-tumorous (A), benign tumorous (B), and malignant tumorous diseases (C). Each dot represents the median of the combined model. The corresponding correlations with RE as the dependent variable are as followed: A, R^2^ = 0.852; B, no significant correlation: C, R^2^ = 0.751. mHAI, histologic activity index.

The multiple linear regression analysis reviled that mHAI remained a significant influence factor in patients with non-tumorous (Fig 6A, p = 0.015) and malignant tumorous diseases (Fig 6C, p = 0.015) with a higher R^2^ in patients with non-tumorous (R^2^ = 0.852) compared to malignant tumorous diseases (R^2^ = 0.751). In patients with benign tumorous diseases (Fig 6B) no significant correlation was observed.

## Discussion

Inflammation and fibrosis are strongly associated: hepatic inflammation initiates fibrogenesis and maintains itself by promoting proinflammatory signaling molecule release and can occur at any stage of liver fibrosis/cirrhosis [1, 2]. To be able to initiate an adequate treatment as early as possible, researchers aim to distinguish inflammation and fibrosis in MR imaging approaches. Variable MR techniques were applied to evaluate the fibrotic and inflammation status of hepatic parenchyma [15]. Based on liver stiffness and damping ratio, assumptions of the NAFLD status and the distinction to initial liver fibrosis have been shown [16, 17]. Similarly, liver kinetics assessed in contrast-enhanced MR imaging as well as the apparent diffusion coefficient can indicate liver fibrosis and inflammation [18–22].

In this study, we analyzed histopathological hepatic inflammation and fibrosis parameters in comparison to signal intensity indices on contrast-enhanced MRI, to analyze the influence of hepatic inflammation on Gd-EOB-DTPA uptake into the liver parenchyma.

Similar to various studies [12, 23–25], we revealed the level of fibrosis as a strong predictor of signal intensity-based indices (p < 0.001). Gd-EOB-DTPA is a hepatocyte-specific MR contrast agent having OATP1-mediated uptake and MRP2-mediated biliary excretion [26]. Hepatobiliary MR contrast agents can be used to characterize the functional properties of the liver parenchyma, and RE might be a reflection of the dysfunction of hepatocytes as a result of the accumulation of liver fibrosis and increased necro inflammatory activity [27].

The development of liver cirrhosis is characterized by destructed lobular and vascular architecture which may promote nodular regeneration of liver tissue. In histological analysis, fibrosis is characterized by an accumulation of extracellular collagen fibers, proteoglycans, and other macromolecules [13]. Since the hepatic uptake of Gd-EOB-DTPA depends on the integrity of the hepatocyte mass, a reduced signal intensity during the HBP and relative enhancement can be assumed. In plain SI-images there is currently a controversy discussion about the influence of liver fibrosis/cirrhosis. Studies showed that the plain SI could be increased due to tissue remodeling in liver fibrosis with inflammation and resulting edema [28–30], as well as reduced based on increased deposition of paramagnetic macromolecules such as collagen tissue with a lower water content [31, 32]. In this study, patients showed rather low levels of inflammation (Fig 3), so one can assume that the influence of fibrotic/cirrhotic tissue remodeling is more dominant than changes during hepatic inflammation.

Nevertheless, significant correlations between hepatic enhancement and signal intensity-based indices could be found. Only a few studies analyzed hepatic inflammation in comparison to contrast-enhanced MRI: Yamada et al. [18] and Tsuda et al. [20] tested in animal studies the effect of NASH caused inflammation on dynamic MR liver kinetics. They observed a significant correlation of the parameters of Gd-EOB-DTPA kinetics (Tmax, T1/2) with steatosis and inflammation [18] and distinct values of time to maximum RE for NASH with grade 2 necroinflammatory activity and control liver (p < 0.01) [20]. A study by Wu et al. [33] revealed a significant difference between NASH and simple steatosis (p = 0.03), a negative correlation of fibrosis stage and RE (r = - 0.469, p = 0.018) and no significant correlation between RE and necroinflammatory grade (p = 0.09). In accordance with the observations of Wu et al., we found a significant correlation between RE and the fibrosis stage (p < 0.001). However, whereas Wu et al. could not find a correlation between the inflammation and RE, we found a significant correlation between inflammation and RE (p = < 0.001). Our study protocol diverged from Wu et al., as we used the scoring system according to Ishak [13, 14], a more detailed scoring system not only for liver fibrosis but also for hepatic inflammation, while Wu et al. interpreted their samples by the Brunt classification, a grading system in the case of nonalcoholic steatohepatitis [34]. In addition, Wu et al. investigated the effects of hepatic inflammation in fatty liver diseases, whereas we studied a mixed population regarding the influence of hepatic inflammation on contrast enhancement.

In the development of inflammation and fibrosis, various factors such as oxidative stress, mitochondrial changes and hormonal disorders are considered as determinants [35–37]. Inflammation plays an important role in the remodeling of liver parenchyma [28–30]. In response to damaged hepatocytes, apoptotic bodies are recruited to interact with dormant hepatic stellate cells and Kupffer cells to activate and promote inflammatory and fibrogenic reactions [38, 39]. Consequently, the enhanced inflammatory and immune-mediated responses will promote hepatocyte necrosis and apoptosis, which promotes further fibrogenic processes [40]. The subgroup analysis of the mHAI criteria revealed that the status of portal inflammation (mHAI D) and periportal or periseptal interface hepatitis (piecemeal necrosis) (mHAI A) are significant predictors (p ≤ 0.001) of RE and the signal intensity of the HBP and the plain images, while focal (spotty) lytic necrosis, apoptosis and focal inflammation (mHAI C) showed a significant correlation with the HBP. As loss and degeneration of hepatocytes characterize piecemeal necrosis at the lobular-portal interface [41], affected hepatocytes will have decreased capacities of gadolinium-chelate-uptake and might show decrease hepatocyte enhancement. Another characteristic of inflammation are lymphocytes which spill into the hepatic parenchyma and the expansion of the extracellular space in the tissue due to increased extracellular fluid accumulation (edema) [42, 43], and thus not only influencing the contrast enhanced images but also the plain images.

Our study has several limitations. Hepatic enhancement analysis was performed with the mean of several distinct ROI measurements to diminish the influence of heterogeneous distributed parenchymal changes. In contrast to this, histopathological samples were received from distinct areas which might not represent hepatic status as accurate as desired. This might also explain the fact that there is a difference regarding the tissue sample. Discrepancies in the evaluation might be possible especially for the subgroup analysis concerning the mHAI criteria. The results of our study support Feier et al.'s statement that strict criteria must be applied to liver biopsy samples when used as reference procedures [44].

In addition, it must be stressed that we analyzed a mixed patient cohort, as it is typical in clinical routine. A homogeneous cohort would be theoretically preferable and more suitable to investigate the different aspects of inflammation. In subgroup analysis (Table 8, Figs 5 and 6) we investigated the influence of hepatic inflammation on parenchymal enhancement for patients with known parenchymal and tumor diseases. This subgroup analysis revealed that the information about hepatic inflammation contributes to the variance elucidation especially in patients with known inflammatory liver parenchymal diseases (R^2^ = 0.849) and non- tumorous diseases (R^2^ = 0.852). Further, prospective studies are needed to investigate heterogeneous etiologies of liver injury, in a retrospective study with only one center, it was difficult to avoid bias in the sampling.

In general, fibrosis and inflammation are complex processes, which are influenced and characterized by several different factors. This study underlines that not only the change in liver architecture associated with hepatic fibrosis / cirrhosis affects uptake, but also other processes such as increased deposition of hepatic water content, hypercellularity and an increased amount of free bound water in the hepatic parenchyma often associated with inflammation of the liver, affect the accumulation of hepatobiliary MR contrast agents such as Gd-EOB-DTPA. Therefore, evaluation and grading based on a single aspect might be insufficient. Due to histological changes which occur in the process of inflammation, an effect on hepatocyte functionality and ability in gadolinium-chelate uptake was expected and could be shown in our study. In conclusion, the level of inflammation and the degree of fibrosis affect the hepatic enhancement indices signal intensity and relative enhancement.
